# Esophageal Cancer in a Patient With Poland Syndrome

**DOI:** 10.31486/toj.25.0036

**Published:** 2025

**Authors:** Glen D. Myatt, Kyle Finney, Vani Vijayakumar

**Affiliations:** ^1^ University of Mississippi School of Medicine, Jackson, MS; ^2^ Department of Radiology, Ochsner Clinic Foundation, New Orleans, LA

**Keywords:** *Adenocarcinoma of esophagus*, *congenital*, *esophageal neoplasms*, *gastrointestinal neoplasms*, *Poland syndrome*

## Abstract

**Background:**

Poland syndrome is an uncommon congenital condition marked by the absence of major chest muscles, typically on one side. Individuals with congenital abnormalities such as Poland syndrome are at increased risk for cancer.

**Case Report:**

A 53-year-old male with undiagnosed Poland syndrome initially presented with dysphagia. The patient had a 20 pack-year smoking history but had quit smoking at the time of presentation. Endoscopic ultrasound showed a large obstructing mass in the distal third of the esophagus, and computed tomography (CT) scan showed an esophageal mass and congenital absence of the left pectoralis major and pectoralis minor muscles. Positron emission tomography-CT (PET-CT) showed a hypermetabolic mass with no evidence of distant metastases. The patient was treated with radiation, chemotherapy, and distal esophagectomy. PET-CT obtained nearly a year postoperatively showed no esophageal mass but some reactive hilar and mediastinal lymph nodes. The patient is monitored annually via CT imaging for recurrent or metastatic disease.

**Conclusion:**

While Poland syndrome has been associated with various malignancies, to our knowledge, the occurrence of esophageal adenocarcinoma in a patient with Poland syndrome has not been previously reported.

## INTRODUCTION

Poland syndrome is an uncommon congenital condition characterized by the unilateral absence of the sternocostal head of the pectoralis major muscle and hypoplasia of the pectoralis minor muscle. Other abnormalities such as rib aplasia, pectus excavatum, digital anomalies, and ipsilateral breast hypoplasia are also associated with Poland syndrome.^1,2^ The estimated incidence is 1 in 10,000 to 1 in 100,000 newborns,^3^ but the condition may be underdiagnosed because mild cases without hand involvement may never come to medical attention.^2^

The cause of Poland syndrome is not well understood, but one hypothesis for its pathogenesis is subclavian hypoperfusion during embryogenesis.^4^ The disruption of blood flow is thought to occur at about the sixth week of embryonic development and affect blood vessels that will become the subclavian and vertebral arteries on each side of the body.^4^ These arteries normally supply blood to embryonic tissues that develop into the chest wall and hand on their respective sides.^5^ Variations in the site and extent of the disruption may explain the range of signs and symptoms that occur in Poland syndrome. Poland syndrome is thought to be a random, sporadic event, but some cases show a familial, autosomal dominant pattern.^6^ Additionally, some cases highlight a teratogenic association with Poland syndrome, as cases of maternal smoking and cocaine use have been associated with the development of this condition.^7,8^

Cases are commonly unilateral and right-sided, with males more frequently affected than females.^2,9^ Various malignancies have been reported in patients with Poland syndrome, but to our knowledge, no cases in the literature have reported a patient with Poland syndrome and esophageal adenocarcinoma.

## CASE REPORT

A 53-year-old male presented to our institution's gastroenterology clinic with recurrent symptoms of dysphagia. The patient's dysphagia symptoms had worsened during the prior 2 months, leading him to make an appointment with his primary care provider. The patient was a 20 pack-year smoker who had stopped smoking shortly before presentation. Family history was noteworthy for breast cancer in his mother and prostate cancer in his father.

Physical examination was negative for abdominal tenderness to palpation; for neurologic weakness; and for head, ear, eye, nose, and throat abnormalities.

**Figure 1.**
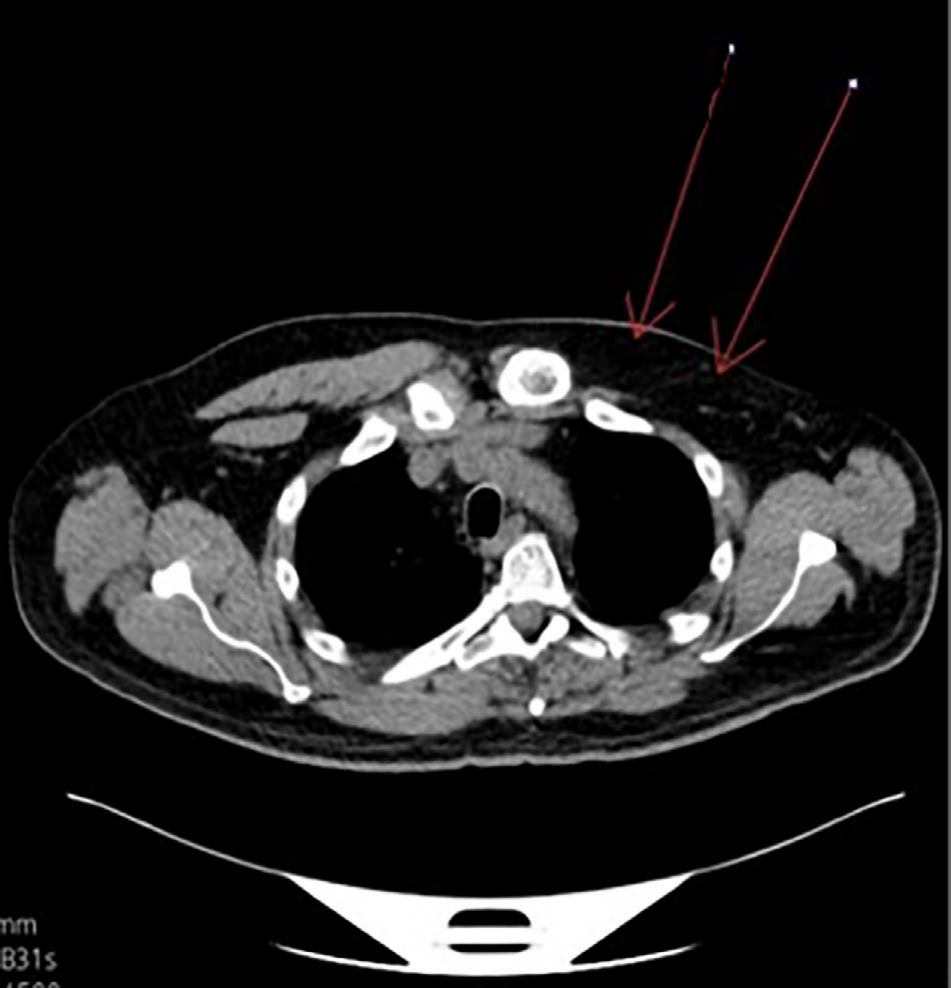
Noncontrast transaxial computed tomography scan shows the absence of the left pectoralis major and pectoralis minor muscles (arrows).

Endoscopic ultrasound of the esophagus demonstrated a large, obstructing, ulcerative mass in the distal third of the esophagus. Biopsy results showed poorly differentiated esophageal adenocarcinoma with signet ring cells. Computed tomography (CT) scan showed an esophageal mass and congenital absence of the left pectoralis major and pectoralis minor muscles, indicating Poland syndrome (Figure 1). The patient had not previously been diagnosed with Poland syndrome.

**Figure 2.**
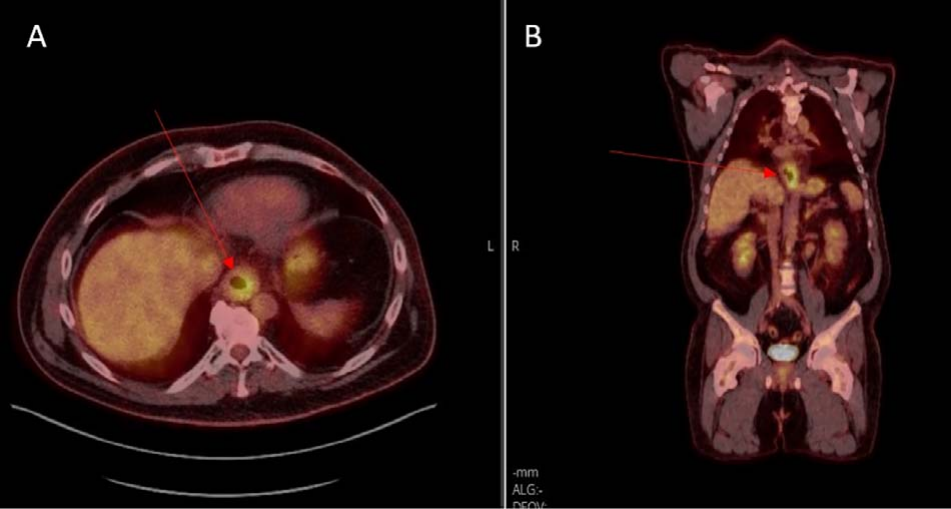
(A) Axial fused positron emission tomography-computed tomography (PET-CT) shows the hypermetabolic mass (arrow) at the distal esophagus (standardized uptake value maximum 18). (B) Coronal fused PET-CT shows the hypermetabolic mass (arrow) at the distal esophagus and extending to the gastroesophageal junction. The patient had regional lymphadenopathy but no evidence of distant metastases.

**Figure 3.**
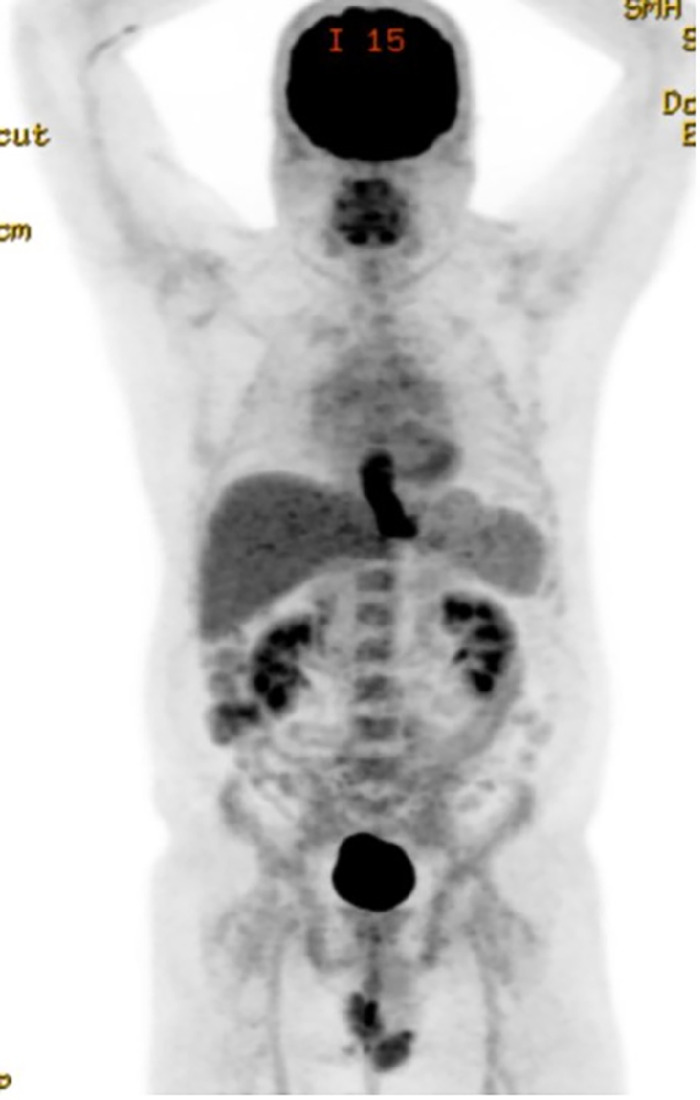
Maximum intensity projection positron emission tomography-computed tomography imaging shows a large hypermetabolic esophageal mass at the distal esophagus and gastroesophageal junction.

Positron emission tomography-CT (PET-CT) scan showed a hypermetabolic mass (standardized uptake value maximum of 18) at the distal esophagus and extending to the gastroesophageal junction. The patient had regional lymphadenopathy but no evidence of distant metastases (Figure 2). Maximum intensity projection (MIP) PET-CT imaging obtained at the time of diagnosis showed a large hypermetabolic esophageal mass at the distal esophagus and gastroesophageal junction (Figure 3).

The patient underwent radiation therapy with 5040 cGy over 28 fractions, as well as 240 mg of nivolumab immunotherapy every 2 weeks for 3 months. After completing neoadjuvant therapy, the patient underwent robotic total thoracic esophagectomy at the gastroesophageal junction with placement of a jejunostomy tube. Pathology specimens demonstrated good response to neoadjuvant treatment with near-complete response to the radiation and chemotherapy. The treatment bed (6.3 cm) consisted of marked mucosal ulceration, fibrosis, and mucin pools with scattered, rare, poorly differentiated tumor cells present in the adventitia. The tumor-node-metastasis description was y. The 23 lymph nodes that were excised showed no signs of tumor invasion. Resected margins were negative for invasive carcinoma, dysplasia, and intestinal metaplasia.

Forty-three days after surgery, the patient began tolerating oral feeding, and the jejunostomy tube was removed. Two months after surgery, the patient was started on a 480-mg nivolumab maintenance dose that was administered every 4 weeks for 1 year.

**Figure 4.**
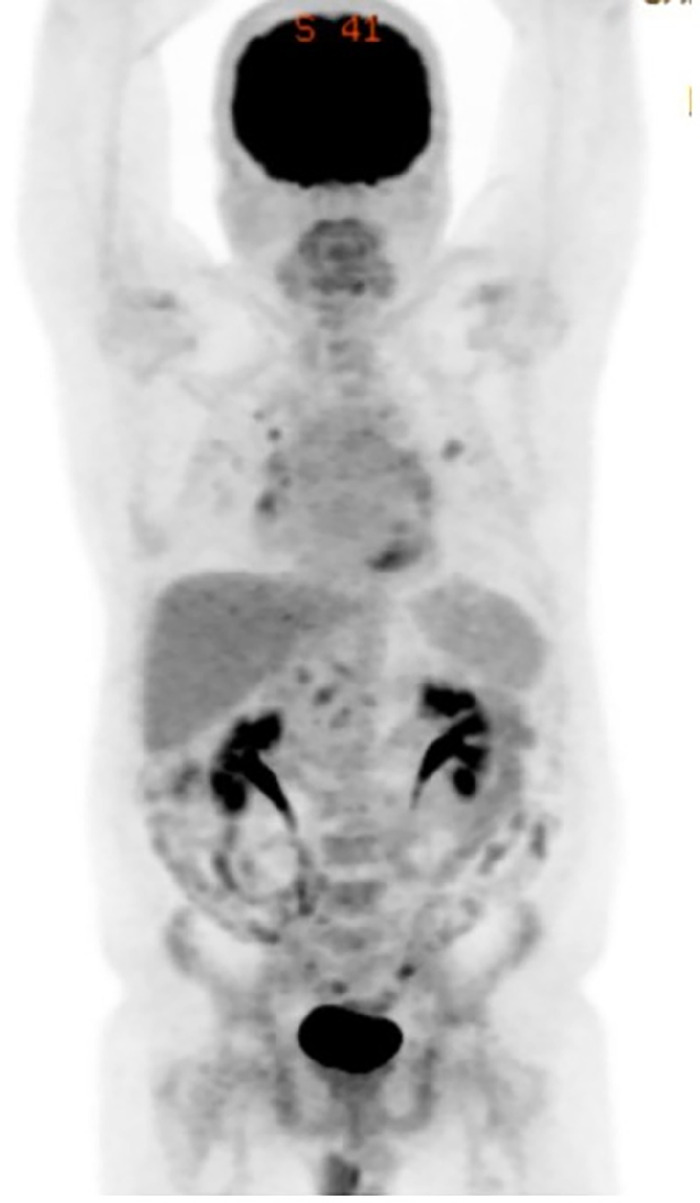
Maximum intensity projection positron emission tomography-computed tomography imaging obtained nearly a year postoperatively shows no esophageal mass but does show some reactive hilar and mediastinal lymph nodes.

Repeat MIP PET-CT obtained nearly a year postoperatively showed no esophageal mass but did show some reactive hilar and mediastinal lymph nodes (Figure 4). The patient is followed with annual CT imaging of his chest and abdomen for surveillance of recurrent and metastatic disease, per American Gastroenterological Association guidelines.^10^

## DISCUSSION

Smoking and family history likely played a role as risk factors in the development of our patient's cancer. He had a 20 pack-year smoking history, and smoking is associated with increased risk for esophageal cancer.^11^ Additionally, the family history of cancer in his mother and father both increased the patient's risk of developing cancer. A history of cancer in parents increases the risk of cancer in their children.^12,13^

In addition to our patient's smoking and familial risk factors, Poland syndrome was another risk factor. Individuals with congenital abnormalities, including nonchromosomal abnormalities such as Poland syndrome, have an increased risk of developing cancer into adulthood,^14^ and various malignancies have been reported in patients with Poland syndrome. Wang et al described the case of a 61-year-old male who developed a lung adenocarcinoma on the same side as his pectoralis muscle defect.^15^ Shaham et al described the case of a 56-year-old female with Poland syndrome who developed high-grade leiomyosarcoma of the right anterior pelvic wall.^16^ Loharkar et al reported the case of a 44-year-old male with Poland syndrome who developed gastric adenocarcinoma.^17^ Guo et al presented the case of a 47-year-old female who developed breast cancer on the same side as her anatomic abnormality.^18^ Hematologic malignancies, including leukemia,^19^ lymphoma,^20^ Wilms tumor,^21^ and neuroblastoma,^22^ have also been reported in patients with Poland syndrome.

For some patients, Poland syndrome abnormalities altered their cancer treatment. In the Guo et al case, the patient with breast cancer required a modified mastectomy because the absence of her right pectoralis major and pectoralis minor muscles and an adhesion band on her lateral chest wall necessitated changes to the standard surgical approach.^18^ Gerlinger et al reported the case of a patient with Poland syndrome who developed a malignant tonsil-lingual tumor that metastasized to the neck.^23^ The absence of pectoralis muscles posed a challenge for reconstruction following tumor excision and radical neck dissection. In this case, the surgeon, who typically preferred using a pectoralis major myocutaneous flap instead opted for a radial forearm free flap.^23^ These cases highlight the importance of understanding that the anatomic variations associated with Poland syndrome can alter standard approaches in cancer treatment.

## CONCLUSION

Patients with congenital abnormalities such as Poland syndrome are at an increased risk for developing cancer. While no association between Poland syndrome and esophageal adenocarcinoma has been established, this case documents the occurrence of esophageal adenocarcinoma in a patient with Poland syndrome. To our knowledge, the occurrence of esophageal cancer in a patient born with Poland syndrome has not previously been reported.

## Notes

*This article meets the Accreditation Council for Graduate Medical Education and the American Board of Medical Specialties Maintenance of Certification competencies for Patient Care and Medical Knowledge.*
